# Factor XII (Hageman) Levels in Women with Recurrent Pregnancy Loss

**DOI:** 10.1155/2014/459192

**Published:** 2014-11-12

**Authors:** A. Seval Ozgu-Erdinc, Cihan Togrul, Ayla Aktulay, Umran Buyukkagnici, Elif Gul Yapar Eyi, Salim Erkaya

**Affiliations:** ^1^Department of Obstetrics and Gynecology, Zekai Tahir Burak Women's Health Care, Training and Research Hospital, Talatpasa Bulvari, 06230 Ankara, Turkey; ^2^Department of Obstetrics and Gynecology, Diyarbakır Women's Health and Children Disease Hospital, Diyarbakır, Turkey; ^3^Department of Biochemistry, Zekai Tahir Burak Women's Health Care, Training and Research Hospital, Talatpasa Bulvari, 06230 Ankara, Turkey

## Abstract

*Objective*. To evaluate factor XII levels in women with recurrent pregnancy loss (RPL) in a tertiary referral hospital. *Methods*. Women who were referred to our hospital for two consecutive abortions or three abortions in between 2007 and 2013 were included in this retrospective observational study. Women were further grouped according to factor XII levels, as <60% and ≥60%. *Results*. Mean factor XII level was 109.1 ± 35.7% (range: 9–200). Ninety-three (7.4%) women had factor XII levels < 60%. Mean factor XII level was 44.8 ± 13.1, and levels ranged between 9 and 60 in this group. Only one woman had factor XII level < 10 %. Remaining 1164 (92.6%) women had factor XII levels ≥ 60%. Mean factor XII level was 114.3 ± 31.7, and levels ranged between 60.3 and 200 in this group, while 1015 (72.4%) women had factor XII levels within the normal range (60%–150% [100% = 30 *μ*g/mL]). *Conclusion*. Decreased activity of F-XII was diagnosed in 7.4% of women with RPL. We concluded factor XII deficiency that might be a rare but significant factor for RPL, and should be evaluated in women who are investigated for recurrent pregnancy loss.

## 1. Introduction

Coagulation and fibrinolysis balance plays an important role in sustaining a normal placental function. Thrombosis of spiral arteries and the intervillous space on the maternal side of the placenta can impair adequate placental perfusion. Impairments in uteroplacental circulation may cause fetal loss, fetal growth restriction, and/or preeclampsia.

Coagulation factor XII (F-XII), which is also known as Hageman factor, is a plasma protein and a single-chain zymogen form of F-XIIa with no detectable enzymatic activity [1]. Factor XII is synthesized in the liver with a molecular weight of 80–90 kD and plasma concentrations are about 30 mg/mL [1]. Factor XII has a major role in the initiation of the intrinsic pathway of blood coagulation. It activates FXI and prekallikrein in the intrinsic pathway of the coagulation cascade. FXII deficiency is rare and may present as an autosomal recessive or acquired disorder. It is generally asymptomatic. On the contrary, excessive F-XII levels may result in venous thrombosis. Previously many studies suggested an association between factor XII (FXII) deficiency and autoantibodies to FXII and recurrent pregnancy losses [2–5]. In this study, we aimed to evaluate the prevalence of F-XII deficiency in women who were investigated for recurrent pregnancy loss (RPL) in a tertiary referral hospital.

## 2. Methods

A cross-sectional study was conducted at Zekai Tahir Burak Women's Health Care, Training and Research Hospital after acquiring approval from the institutional review board. Women who were referred to our hospital for two consecutive abortions or three abortions in between 2007–2013 were included in this retrospective observational study. Pregnancy losses after 12 weeks of gestation were not included in the study. Cases in which F-XII levels could not be measured were excluded. Factor XII activity was measured by the APTT method using a Behring Coagulation System (BCS; Dade Behring Inc., Liederbach, Germany). FXII-deficient plasma (George King Bio-Medical Inc., St. Overland Park, KS, USA) was incubated with Pathromtin SL (Dade Behring Inc.) and 0.02 mol/L CaCl_2_ (Calcium Chloride) (Sysmex International Reagents Co., Ltd., Kobe, Japan). The mixture was incubated with patient plasma or standard plasma and the clotting time was recorded. Activity was expressed as a percentage with reference to the curve for standard human plasma. Women were further grouped according to F-XII cut-off levels 10, 35, 60, and 150 [3, 6]. Statistical analyses were performed with SPSS for Windows 21.0 software. Continuous variables were expressed as mean ± standard deviation (SD), and categorical variables were expressed as number (percentage).

## 3. Results

Within the study period, 1401 women were assessed for F-XII levels. Among these, 144 women were excluded because of technical unavailability of F-XII measurements. A total of 1257 women were eligible for final analysis. Mean patient age was 29.6 ± 9.2 years (range 18–39). Median abortion count was 2 (range 2–4) (Table 1). Mean F-XII level was 109.1 ± 35.7% (range 9–200) (Table 2). Ninety-three (7.4%) women had F-XII levels <60%. Mean F-XII level was 44.8 ± 13.1, and levels ranged between 9 and 60 in this group. Only 1 woman had F-XII level <10% and 20 women had F-XII levels <35% which makes the F-XII deficiency prevalence of our study population 1.7%. Remaining 1164 (92.6%) women had F-XII levels ≥60%. Mean F-XII level was 114.3 ± 31.7, and levels ranged between 60.3 and 200 in this group.

## 4. Discussion

Recurrent pregnancy loss may be associated with disturbances in coagulation and fibrinolysis cascade. In a recent study from Greece, FXII deficiency and the number of abortions in the recurrent abortion group were found to be correlated [6]. In previous studies the prevalence of F-XII deficiency has been reported between 2.9 and 15% of women with RPL [3, 6–8]. Also in Matsubayashi et al.'s study [9], decreased activity of F-XII was found to be associated with recurrent in vitro fertilization failure. There have been previous reports of an association of FXII deficiency with RPL [2–5] and in a larger study of 500 women with unexplained primary recurrent miscarriages [10]; F-XII deficiency and hypofibrinolysis were found to be the most frequent abnormalities of the hemostatic system [11].

The presence of antibodies to factor XII has been shown to lead to F-XII deficiency [4]. In the routine setting neither factor XII levels nor antibodies against factor XII are evaluated. The etiology could not be explained in the half of patients with recurrent pregnancy loss. Presence of antibodies to factor XII and factor XII deficiency may help to reveal a cause for unexplained RPL.

In this study, we investigated F-XII levels in women with RPL. In our study 7.4% of women with RPL had reduced F-XII activity, while F-XII deficiency was present in only 1.7%. Therefore, we conclude that although FXII deficiency might be a rare but significant factor for RPL, and should be evaluated in women who are investigated for recurrent pregnancy loss.

## Figures and Tables

**Table 1:** Characteristics of the study population.

		Min–max
Age, years (mean ± SD)	29.6 ± 9.2	18–39
Gravidity (median)	4	1–12
Pregnancy loss (median)	2	2–10
Previous live birth (median)	0	0–2

**Table 2:** Distribution of factor XII levels among patients with recurrent pregnancy loss.

	*n*	%	Mean ± SD
<10%	1	0,1	9
10–35%	20	1,6	25,85 ± 6,39
35–60%	73	5,8	50,81 ± 7,73
60–150%	1014	80,7	105,53 ± 22,38
150–200%	149	11,9	174,50 ± 17,47

Total	1257	100	109,19 ± 35,74
